# Evaluation of the efficiency of nanomicellar formulation of fat-soluble vitamins in patients with cystic fibrosis: the study protocol for a randomized controlled trial

**DOI:** 10.1186/s13063-023-07896-8

**Published:** 2024-01-16

**Authors:** Mahsa Soleimanzadeh, Saeedeh Talebi, Mahmoud Reza Jaafari, Seyed Javad Sayedi, Maryam Emadzadeh, Hamid Reza Kianifar

**Affiliations:** 1 Faculty of Medicine, Mashhad University of Medical Sciences, Mashhad, Iran; 2 Department of Nutrition, Faculty of Medicine, Mashhad University of Medical Sciences, Mashhad, Iran; 3 Nanotechnology Research Center, Pharmaceutical Technology Institute, Mashhad University of Medical Sciences, Mashhad, Iran; 4 Department of Pediatrics, Mashhad University of Medical Sciences, Mashhad, Iran; 5 Clinical Research Development Unit, Ghaem Hospital, Mashhad University of Medical Sciences, Mashhad, Iran

**Keywords:** Cystic fibrosis, Pancreatic insufficiency, Malabsorption, Fat-soluble, Vitamins, Nanomicelle, Children, Randomized controlled trial

## Abstract

**Background:**

Cystic fibrosis is an inherited disease, which is caused by the CFTR protein defects due to mutations in the CFTR gene. Along with CFTR dysfunction, exocrine pancreatic insufficiency plays a key role in persistent fat malabsorption in CF patients; therefore, deficiency of fat-soluble vitamins (A, D, E, and K) is still a therapeutic challenge. Even with efficient pancreatic enzyme medication and CF-specific vitamins, many patients with CF have fat-soluble vitamins deficiency. The present study aims to evaluate the efficiency of nanomicelle formulation of fat-soluble vitamins in children with CF in order to achieve the appropriate serum levels of these vitamins.

**Methods:**

This prospective, single-blind control trial will be conducted at the Akbar Children’s Hospital in Mashhad, Iran. Patients with CF will be enrolled based on the eligibility criteria. The control group will receive the standard formulation of fat-soluble vitamins similar to the routine CF treatment, and for the intervention group, the nanomicelle formulation of fat-soluble vitamins will be administered for 3 months. The primary outcome of this study is the measurement of serum levels of fat-soluble vitamins. The secondary outcomes are clinical assessment by the Shwachman-Kulczycki score, anthropometrics, and quality of life. Outcomes will be assessed before and after 3 months.

**Discussion:**

Due to persistent fat-soluble vitamin deficiency in CF disease, the nanomicelle formulation could be proposed as a new delivery method of fat-soluble vitamins in the treatment of cystic fibrosis.

**Trial registration:**

Iranian Registry of Clinical Trials IRCT20220415054541N1. Registered on July 23, 2022.

## Administrative information

**Table:** Note: The numbers in curly brackets in this protocol refer to SPIRIT checklist item numbers. The order of the items has been modified to group similar items (see http://www.equator-network.org/reporting-guidelines/spirit-2013-statement-defining-standard-protocol-items-for-clinical-trials/)

Title {1}	Evaluation of the Efficiency of Nanomicellar Formulation of Fat-Soluble Vitamins in Patients with Cystic Fibrosis: The Study Protocol for a Randomized Controlled Trial
Trial registration {2a and 2b}.	Iranian Registry of Clinical Trials IRCT20220415054541N1. Registered on July 23, 2022. For more detailed information on this registration, please refer to https://en.irct.ir/trial/64108
Protocol version {3}	This is the first version of the protocol, 2022-07-23
Funding {4}	The vice chancellery for research of Mashhad University of Medical Sciences (MUMS)
Author details {5a}	1-Faculty of Medicine, Mashhad University of Medical Sciences, Mashhad, Iran. 2-Pediatrician, PhD of Nutrition, Department of Nutrition, Faculty of Medicine, Mashhad University of Medical Sciences, Mashhad, Iran. 3-Nanotechnology Research Center, Pharmaceutical Technology Institute, Mashhad University of Medical Sciences, Mashhad, Iran. 4-Professor of PediatricPulmonology, Department of Pediatrics, Mashhad University of Medical Sciences, Mashhad, Iran. 5-Clinical Research Development Unit, Ghaem Hospital, Mashhad University of Medical Sciences, Mashhad, Iran. 6-Professor of Pediatric Gastroenterology, Department of Pediatrics, Mashhad University of Medical Sciences, Mashhad, Iran.
Name and contact information for the trial sponsor {5b}	For research of Mashhad University of Medical Sciences (MUMS), all study stages will be undertaken under its supervision. Tel: 009838433363Email: ramresearch@mums.ac.ir
Role of sponsor {5c}	All study stages will be undertaken under its supervision.

## Introduction

### Background and rationale {6a}

Cystic fibrosis (CF) is a life-shortening, autosomal recessive genetic disorder, and the most common inherited disease in Caucasians. The disease prognosis has improved considerably in recent decades with the advanced treatments, and the mortality rate has been reduced by 2% annually. The average age of survival in CF patients has been reported to be 65 years in men and 56 years in women [1]. CF is caused by mutations in the cystic fibrosis transmembrane conductance regulator (CFTR) gene, resulting in impaired pancreatic function and, as a result, reduced intestinal absorption of lipids and fat-soluble vitamins, particularly in patients with CF developing pancreatic insufficiency [2]. Fat-soluble vitamins deficiency is still a current therapeutic challenge in CF, chronic pancreatitis, and biliary atresia. A large number of patients exhibit vitamin K or D insufficiencies, and the prevalence of vitamin E and A deficiencies is also significant despite full compliance [3]. In many cases, increases in vitamin dosage are not efficacious, and intractable fat-soluble vitamin deficiencies are diagnosed in CF patients, raising the issue of whether modern drug delivery methods could improve the condition of patients [3, 4]. Although the standard formulation is still used in CF patients, there are new delivery technologies, such as the liposomal form of vitamin E, the nanomicelle form of curcumin, and specific carotenoids [5]. To improve stability and increase the solubility of fat-soluble or poorly water-soluble compounds, several strategies such as liposomal, nanoliposomal, and micellar forms have been recommended. One is using nanomicelles which are efficient tools to encapsulate the drugs with a low aqueous solubility [6]. The nanomicelle has a core-shell structure that prevents the penetration of water in its inner core creating an appropriate environment for the encapsulated drug versus the free drug [6]. Other advantages of micelles as drug carriers include facilitated transport through biological barriers, protection against degradation, being cost-effective, and increased solubility in aqueous media that include the unstirred water layer of the intestine [7]. Consequently, the concept of using the nanomicelle formulation of fat-soluble vitamins could be admirable in CF patients through the facility of absorption of nano-sized vitamins and the lack of necessity of the presence of pancreatic enzymes for their absorption process. In this study, we focused on a novel nanomicelle formulation of vitamins A, D, E, and K, and the main purpose was to address the problem of intractable fat-soluble vitamin deficiencies in pancreatic-insufficient CF patients. The present study aimed to evaluate the efficiency of the novel nanomicelle formulation of fat-soluble vitamins in CF (Table 1).

**Table 1:**
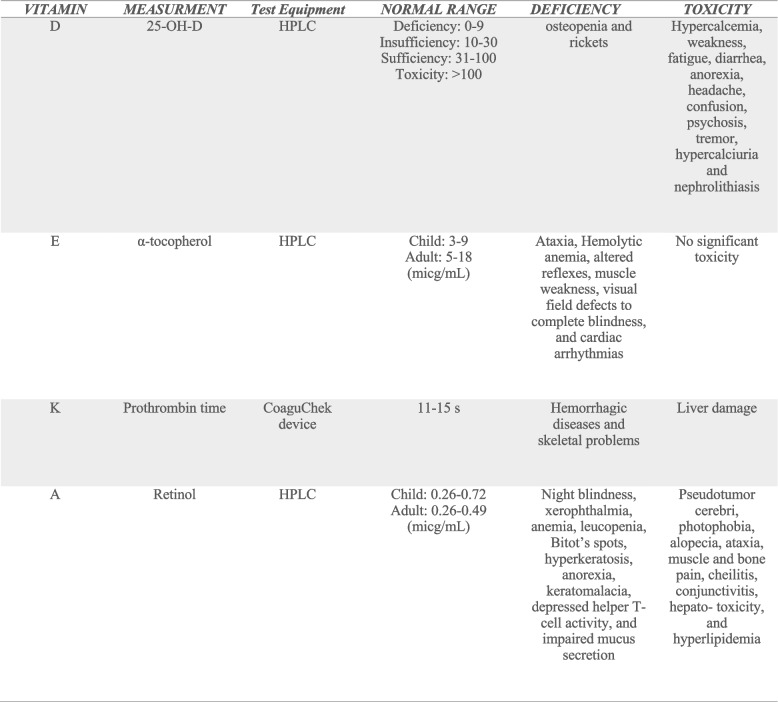
Fat-soluble vitamins—classification, laboratory measurements, test equipment, normal range, deficiencies, and toxicities

### Objectives {7}

The present study aimed to evaluate the efficacy of the novel nanomicelle formulation of fat-soluble vitamins in CF patients to achieve the appropriate serum level of fat-soluble vitamins.

### Trial design {8}

This is a prospective, single-center, parallel, randomized placebo-controlled trial. The study protocol conforms to the Standard Protocol Items Recommendation for Interventional Trials (SPIRIT) Checklist (Table 2).

**Table 2:**
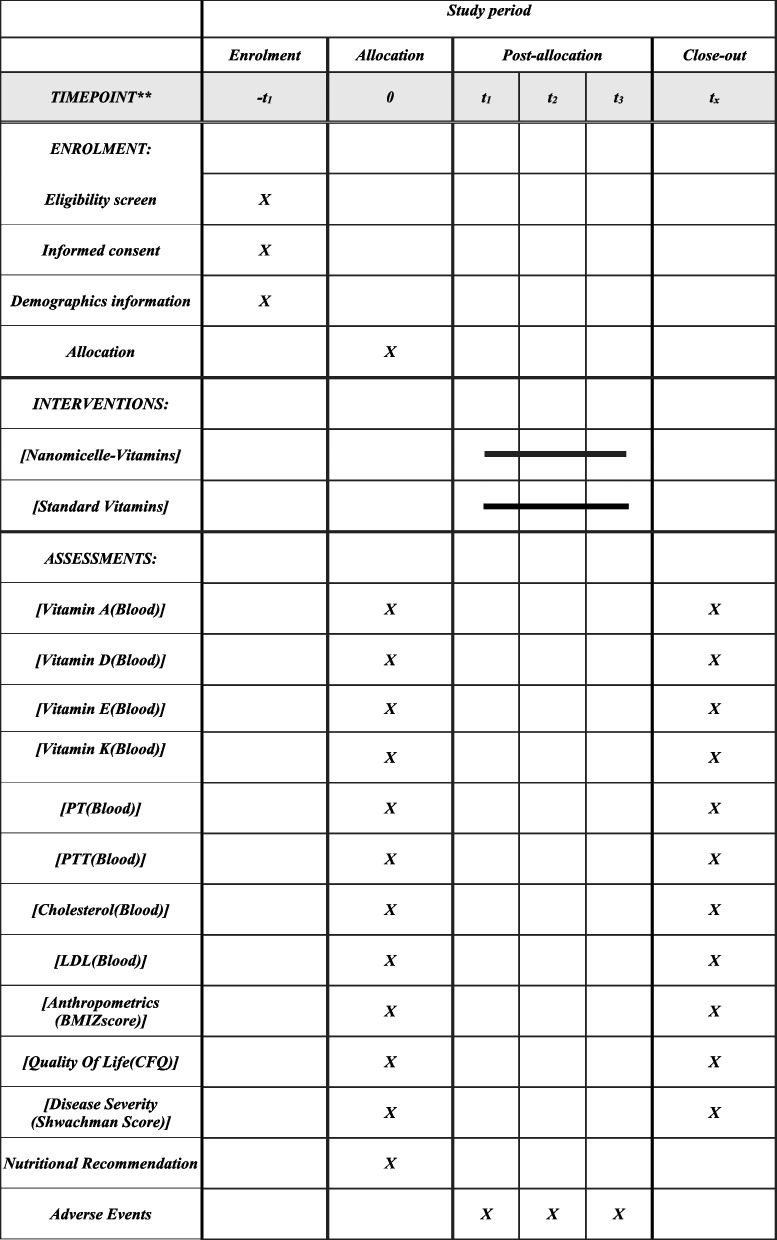
Schedule of enrolment, intervention, and assessment based in the Standard Protocol Items Recommendations for Interventional Trials (SPIRIT) guideline

## Methods: participants, interventions, and outcomes

### Study setting {9}

The study population will comprise the known CF patients referred to the Cystic Fibrosis Clinic at Akbar Children’s Hospital, Mashhad, Iran.

### Eligibility criteria {10}

#### Inclusion criteria

The inclusion criteria of the study include (1) one or more typical phenotypic features of CF and a minimum of an elevated sweat chloride concentration on two/more occasions or two mutations known to cause CF on separate alleles, (2) age ≥ 8 years old, (3) gastrointestinal and pulmonary involvement, (4) the oxygen saturation percentage based on the pulse oximetry of ≥ 90% at room temperature, (5) a clinically stable situation, and (6) informed consent of the patient’s parents.

#### Exclusion criteria

The exclusion criteria of the study include (1) cardiovascular, renal, or hepatic impairment; (2) celiac disease; and (3) no adherence to drug consumption.

### Who will take informed consent? {26a}

Initially, the first researcher will clarify the study objectives to the children’s parents and legal guardians, and written informed consent will be obtained from them at the time of enrolment.

### Additional consent provisions for collection and use of participant data and biological specimens {26b}

In the consent form, the trial members will be inquired whether they concur with utilizing their information in case they withdraw from the trial. Additionally, their consent will be obtained by the research team to share relevant data with the scholarly specialists chosen from the universities or other administrative specialists. Notably, this trial does not include the collection of organic examples for storage.

## Interventions

### Explanation for the choice of comparators {6b}

The participants in the control group will receive the standard formulation of fat-soluble vitamins similar to the routine CF treatment; this is the reason for considering the routine CF treatment as the control group in our study.

### Intervention description {11a}

Nanomicelle fat-soluble vitamins (Exir Nano Sina Drug Company, Iran) are prepared as nanomicelle formation including 1500 units of vitamin A, 3000 units of vitamin D, 150 units of vitamin E, and 1000 μg of vitamin K in 1 cc of the drop. The nanomicelle formulation has been labeled by Exir Nano Sina Company, respectively, and will be administered orally alone or with sucrose-sweetened water once daily at bedtime at a daily dosage of 1 cc for 3 months.

### Criteria for discontinuing or modifying allocated interventions {11b}

Although no side effects have been reported in previous similar studies, in the event of any intervention-related side effects, we will stop the intervention and report the situation to the ethics committee of Mashhad University of Medical Sciences (MUMS) for making decisions [3]. Additionally, if the participants request to discontinue treatment or report the deterioration of their condition due to CF, we will stop the intervention.

### Strategies to improve adherence to interventions {11c}

The strategies include taking the medicine once a day versus several times, before bedtime to minimize interference with other common CF medications, and using an oral drop in comparison to soft gel for easier consumption by children. Individualized training of the patients and their parents will inform them of the necessity of the intervention complement for an optimal impact. If the study members use less than 70% of the drugs, it shows adherence deficits.

### Relevant concomitant care permitted or prohibited during the trial {11d}

CF patients who have a pancreatic deficiency require PERT for enzyme replacement. In this respect, we enrolled CF patients with gastrointestinal involvement; all the patients use CREON as a common treatment in addition to Omeprazole to facilitate maximal effectiveness. Inhaled beta2-agonists are prescribed to CF patients with moderate to severe lung disease twice a week. Also, CF patients who have *Pseudomonas* sp. infection are treated with the inhalation form of tobramycin. Constantly low-dose azithromycin treatment is used in our patients that has an anti-inflammatory effect in CF patients 6 years of age and older.

### Provisions for post-trial care {30}

There is no anticipated harm or compensation for involvement in this trial. However, the follow-up will be carried out via phone calls every 2 weeks to determine if there are any complications.

### Outcomes {12}

The primary outcome of the current trial is the measurement of the serum levels of fat-soluble vitamins once at the onset of enrollment and once at the end of the trial after 3 months.

**Fig. 1:**
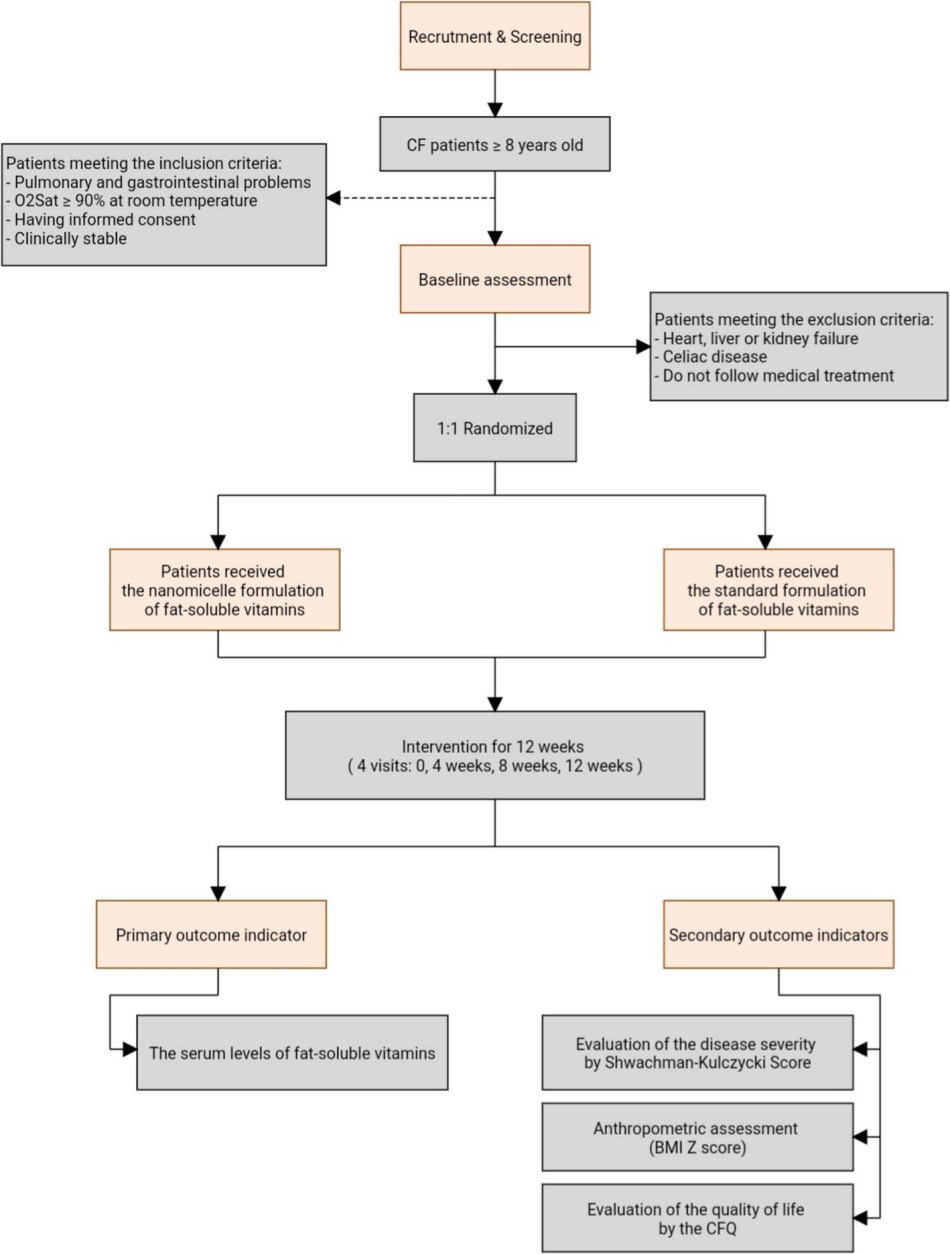
Study design flow chart

The secondary outcomes are clinical criteria evaluation by the Shwachman-Kulczycki score (an index to evaluate the severity of the disease in CF patients, which includes general activity, physical examination, nutrition, and radiological findings), anthropometric assessment by the BMI *Z* score, and quality of life evaluation by using the CFQ (Health-Related Quality of Life in Children with Cystic Fibrosis) from the baseline to 3 months of the intervention (Fig. 1).

### Participant timeline {13}

1. Nutritional suggestions will be given to all the patients and their parents and legal guardians based on the CF requirements and modifiable CREON dosing according to the level of dietary fat. Additionally, the patients or their parents completed the CFQ, depending on age.
2. All anthropometric measurements (weight and height without shoes) will be performed under equal guidance using a digital scale (model: SECA).
3. The primary and secondary outcomes of the treatment will be examined before and after 3 months of treatment. The clinical evaluation and follow-up will be carried out via monthly visits and phone calls with the patients.
4. Approximately 6 mL of blood will be collected before and after 3 months from the beginning of the trial and centrifuged, with the serum separated from the sediment, and preserved at a minimum temperature of − 20 °C.

### Sample size {14}

Due to the lack of clinical trials on the evaluation of the efficacy of nanomicelle formulation of fat-soluble vitamins in children with cystic fibrosis, we use the researcher’s experience to estimate the sample size. According to this, about 60% of CF patients who use the routine formulation of fat-soluble vitamins are treated for vitamin D deficiency. It is estimated that the new method will increase the recovery rate by 30–40%. Considering alpha = 0.05 and power = 80%, the minimum required sample size in each group is estimated to be 33 people. By considering the 10% dropout rate for the sample, a total of 37 samples for each group was calculated. In total, 74 patients with CF are needed to be included in the randomization aspect of this research.

### Recruitment {15}

Our patients will be chosen from the cases referred to the specialized CF clinic at Akbar Children’s Hospital, which covers all the CF patients in our regional state. The initial data is available on the CF registry for easier access. The patients are also supported by the local CF association.

## Assignment of interventions: allocation

### Sequence generation {16a}

The participants of the study who followed the inclusion criteria were divided into two groups: nanomicelle fat-soluble vitamins oral drops as the intervention group and the standard form of fat-soluble vitamins as the control group. Special websites for randomization are used in order to allocate participants into two groups.

### Concealment mechanism {16b}

According to the obtained sequence, the trial researchers will put the codes in the sealed opaque envelopes in order, and when the patients visit the clinic, one envelope is taken in order, and according to the code written in it, the patient is placed in the intervention or control group.

### Implementation {16c}

The main researcher will perform the generation of the allocation sequence, the enrollment of participants, and the assignment of participants to interventions.

## Assignment of interventions: blinding

### Who will be blinded {17a}

Due to the distinguishable appearance of the drugs used in the intervention and control groups, both participants and the main researcher are aware of the group assignments for the patients. However, the data analyst and the nutritionist, responsible for regular communication through weekly phone calls and monthly in-person visits, are blinded to the trial details. In clinical trial studies, the term “blinded” typically applies to patient involvement. In this study, only the data analyst and the nutritionist, who assess patients, operate under blind conditions. Consequently, the trial is registered as “not blinded” in accordance with standard practices in clinical trial blinding.

### Procedure for unblinding if needed {17b}

As mentioned, there is no possibility of blinding researchers and patients due to the type of intervention, so this item is not applicable to this study.

## Data collection and management

### Plans for assessment and collection of outcomes {18a}

As the primary outcome of the present study, the serum level of fat-soluble vitamins will be measured by the laboratory test using the HPLC method. To ensure the quality of the employed laboratory methods, the performance specifications will be verified before using the kits for the analysis of the patient’s samples. All the tests will be carried out in duplicate, and the conventional curve will be prepared using the serial dilutions of the standard samples. The secondary outcomes of the present study are anthropometric assessments, which will be performed in a standard position using SECA instruments for height and weight. Additionally, the WHO growth standard charts will be used for the evaluation of the BMI *Z*-score. The clinical symptoms will be obtained by the Shwachman-Kulczycki score, and the children’s quality of life will be assessed by Health-Related Quality of Life in Children with Cystic Fibrosis to investigate the role of fat-soluble vitamins in the lives of CF patients.

### Plans to promote participant retention and complete follow-up {18b}

Because of the fact that most of the patients are from the pediatric population, we will ask their parents to provide written feedback on the observed outcomes of the evaluations in their children in addition to regular follow-up by phone calls or free-of-charge visits. Also, the nanomicelle fat-soluble vitamins oral drops are provided to the patients at no cost.

### Data management {19}

In the current trial, the database will be created from the questionnaire of clinical data and laboratory assay results in Excel software and SPSS. The trial researchers will collect the data at the beginning and during the follow-up and record the obtained data in the paper checklists as well as the designed Excel datasheet. Next, the data will be analyzed in SPSS. To ensure security, the login password is allowed only for the trial researchers. The database will only accept the variables that are consistent with the previous entries, and it could also be altered in the case of randomly missing values. Semimonthly, 10% of the electronic data will be randomly compared with the paper questionnaires.

### Confidentiality {27}

Participant data, laboratory specimens, collected data, and reports will be identified by a coded ID number. Data collected during the course of the research will remain strictly confidential and only be accessed by the research team. Additionally, the unidentified data will be shared with other researchers to allow international prospective meta-analyses.

### Plans for collection, laboratory evaluation, and storage of biological specimens for genetic or molecular analysis in this trial/future use {33}

This intervention does not involve the storage of biological specimens for genetic or molecular analysis

## Statistical methods

### Statistical methods for primary and secondary outcomes {20a}

SPSS will be used to analyze the data. The Kolmogorov-Smirnov test will be used to evaluate data distribution. The data are expressed as frequencies and percentages for the qualitative variables, the mean and standard deviation for the normally distributed continuous variables, and the median and interquartile range for the other variables. Additionally, intergroup differences will be evaluated using a paired *T*-test for the normally distributed continuous variables and the Wilcoxon rank-sum test for the other continuous variables. Intergroup differences will be assessed using an independent *T*-test for the normally distributed continuous variables and the Mann-Whitney *U*-test for the other quantitative variables. The mean changes in the secondary outcomes will also be adjusted using the analysis of covariance (ANCOVA). The *P* value of less than 0.05 will be considered significant in all the numerical analyses.

### Interim analyses {21b}

In the event of frequent side effects, we will stop the intervention and present the results to the ethics committee of Mashhad University of Medical Sciences (MUMS) for further determination.

### Methods for additional analyses (e.g., subgroup analyses) {20b}

The analysis will be conducted using SPSS software. Assessment of data distribution will involve the Kolmogorov-Smirnov test. Qualitative variables will be presented as frequencies and percentages, while normally distributed continuous variables will be expressed as mean and standard deviation. Other variables will use the median and interquartile range. For intergroup differences, the paired *T*-test will be applied to normally distributed continuous variables, and the Wilcoxon rank-sum test will be used for the other continuous variables. Intragroup differences will be assessed using an independent *T*-test for normally distributed continuous variables and the Mann-Whitney *U*-test for other quantitative variables. Mean changes in secondary outcomes will be adjusted using analysis of covariance (ANCOVA). The *P* value of less than 0.05 will be considered significant in all the numerical analyses.

### Methods in analysis to handle protocol non-adherence and any statistical methods to handle missing data {20c}

To include the outcome data of the participants in the data analysis regardless of protocol adherence, all the analyses will be performed in agreement with the intention-to-treat principle. The last observation by the forward method will be used to impute the missing data for the intention-to-treat analysis.

### Plans to give access to the full protocol, participant-level data, and statistical code {31c}

At the earliest opportunity, we will essay to release the full study protocol and results, irrespective of the magnitude or direction of the effect. The anonymized data set and statistical code may be available from the corresponding author (email: talebis@mums.ac.ir) on reasonable request.

## Oversight and monitoring

### Composition of the coordinating center and trial steering committee {5d}

Due to the low-risk intervention, the data monitoring committee was not involved in the current study. The trial is overseen by a diverse team of healthcare professionals, including gastrointestinal specialists, pulmonologists, nutritionists, and other experts. The blinded nutritionist, unaware of group assignments, maintains regular communication through weekly phone calls and monthly in-person visits, contributing to the ongoing success of the trial. Additionally, the Cystic Fibrosis Clinic actively participates in these regular sessions to monitor progress and maintain the trial’s steady momentum. However, all researchers are committed to reporting any adverse events.

### Composition of the data monitoring committee, its role and reporting structure {21a}

The establishment of a data monitoring committee (DMC) was deemed unnecessary for this particular study due to the low-risk nature of the intervention. The trial is being closely monitored by the ethics committee of Mashhad University of Medical Sciences (MUMS) as part of their oversight role throughout the trial. The ethics committee ensures adherence to ethical guidelines, patient safety, and the integrity of the trial, thereby ensuring the well-being and protection of participants.

### Adverse event reporting and harms {22}

Adverse events were not reported, although potential minor events may occur, which will be reported to the ethics committee of Mashhad University of Medical Sciences (MUMS) for further decision-making.

### Frequency and plans for auditing trial conduct {23}

The trial’s adherence to ethical guidelines will be diligently overseen by the ethics committee of Mashhad University of Medical Sciences (MUMS). They will closely monitor the trial’s integrity and accuracy, commencing from the protocol drafting phase to the conclusion of the entire research process. The committee has set a schedule for regular checks, ensuring at least two comprehensive assessments during the trial to maintain its robustness and adherence to ethical standards.

### Plans for communicating important protocol amendments to relevant parties (e.g., trial participants, ethical committees) {25}

Prior to implementation, all the modifications to the protocol that may affect the conduct of the study will be approved by the ethical committee of MUMS.

### Dissemination plans {31a}

It takes about 4–6 months for the approval and submission of the reports for dissemination via journal publication. Subsequently, the results will be disseminated in spite of the magnitude or direction of their effects. In addition, all the conditions related to publishing the results of the trial pertaining to the corresponding author, and there is no publication limitation enforced by the sponsor.

## Discussion

Vitamins are crucial nutrients that ensure optimal growth, reproduction, and function. They have been classified based on solubility as water-soluble (Bs and C) and fat-soluble (A, D, E, and K) [8]. Fat-soluble vitamins are structurally lipophilic, hydrophobic molecules that are assembled from isoprenoid units. They are soluble mainly in lipids or oils and are thus called fat-soluble vitamins. Absorption and transportation of these vitamins are predominantly associated with lipids in the intestine; therefore, they are absorbed after undergoing mi-cellar solubilization depending on the bile salt and pancreatic enzymes, metabolized and stored in the liver and adipose tissue, and removed slowly from the body owing to their lipophilic character [9]. Abnormal CFTR function in CF patients causes pancreatic ductular obstruction and may result in subsequent pancreatic insufficiency [10]. Pancreatic enzymes and bicarbonate could not be secreted into the duodenum, leading to loss of enzymatic breakdown of nutrients and precipitation of bile salts that cause intestinal malabsorption [11]. Some 85–90% of patients have mutations that are associated with the development of severe pancreatic disease and fat maldigestion (pancreatic insufficiency), and the remaining 10–15% have mutations causing milder pancreatic disease with normal fat digestion (pancreatic sufficiency) [12]. Since CF is the most common inherited life-shortening disease and about 90% of CF patients in the US have pancreatic insufficiency and fat-soluble vitamin deficiency, in spite of using effective pancreatic enzyme medications and CF-specific vitamin and mineral supplements, they may require new forms of vitamins to address the deficiency [13–19]. Consequently, new delivery methods such as emulsions, nanoemulsions, nanomicelles, liposomes, hydrogels, and lipid nanoparticles could be utilized as appropriate formulations for the delivery of fat-soluble vitamins to CF patients and others with pancreatic insufficiency [3, 20]. Recently, the nanomicelle formulation of fat-soluble vitamins has been studied, considering its impact on the increased bioavailability of fat-soluble vitamins. The main limitation of this study is the dissimilarity between the forms of drugs in the two groups. While in the nanomicelle group, patients take only one dose per day; in the control group, they take four pills daily. To the best of our knowledge, this is the first study that will assess the use of nanomicelle formulation of fat-soluble vitamins in CF patients.

## Trial status

Recruitment started on July 24, 2022, and completed on November 30, 2022. The trial procedures are expected to be completed by the end of April 2023. The last version of the protocol was issued on July 23, 2022.

## Data Availability

The datasets generated and analyzed during the current study are not publicly available due to ethical considerations, but they may be available from the corresponding author on reasonable request.

## Abbreviations

BMI: Body mass index
CF: Cystic fibrosis
CFQ: Cystic fibrosis questionnaires
CFTR: Cystic fibrosis transmembrane conductance regulator
RCT: Randomized control trial
PERT: Pancreatic enzyme replacement therapy
HPLC: High-performance liquid chromatography
ANCOVA: Analysis of covariance
